# Anxiety and hemodynamic reactivity during cardiac stress testing: The role of gender and age in myocardial ischemia

**DOI:** 10.1007/s12350-020-02079-3

**Published:** 2020-02-28

**Authors:** Maria T. Bekendam, Paula M. C. Mommersteeg, Willem J. Kop, Jos W. Widdershoven, Ilse A. C. Vermeltfoort

**Affiliations:** 1 Department of Medical and Clinical Psychology, Center of Research on Psychology in Somatic Diseases (CoRPS), Tilburg University, Warandelaan 2, P.O. Box 90153, 5037 AB Tilburg, The Netherlands; 2 Department of Nuclear Medicine, Institute Verbeeten, Tilburg, The Netherlands; 3 Department of Cardiology, Elizabeth-TweeSteden Hospital, Tilburg, The Netherlands

**Keywords:** Age, Cardiac stress testing, Myocardial ischemia, Sex differences, Trait anxiety

## Abstract

**Background:**

The prevalence of myocardial ischemia is associated with anxiety. State and trait anxiety are more common in younger women compared to men, and high anxiety levels could affect hemodynamic reactivity during cardiac stress testing. The aim is to examine whether anxiety plays a role in gender differences in patients ≤ 65 and > 65 years in hemodynamic reactivity and ischemia during cardiac stress testing.

**Methods and results:**

Included were 291 patients (66.8 ± 8.7 years, 45% women) with suspect ischemia undergoing myocardial perfusion single-photon emission computed tomography (MPI-SPECT). Primary outcomes were semi-quantitative summed difference score (SDS) and summed stress score (SSS), as continuous indicators of myocardial ischemia. Analyses were stratified by age. Trait anxiety was measured using a validated questionnaire (GAD-7) and state anxiety using facial expression analyses software. Overall, trait and state anxiety were not associated with the prevalence of ischemia (N = 107, 36%). A significant interaction was found between gender and trait anxiety in women ≤ 65 years for SDS (*F*(1,4) = 5.73, *P *= .019) and SSS (*F*(1,10) = 6.50, *P *= .012). This was not found for state anxiety.

**Conclusion:**

SDS and SSS were significantly higher in women younger than 65 years with high trait anxiety. This interaction was not found in men and women over 65 years.

**Electronic supplementary material:**

The online version of this article (10.1007/s12350-020-02079-3) contains supplementary material, which is available to authorized users.

## Introduction

Traditional cardiovascular risk factors (e.g., smoking, hypertension, and diabetes mellitus) are clinically important but do not fully account for the gender differences in the increasing rates of cardiovascular risk and hospitalizations in younger women.^1^,^2^ Large epidemiological studies^3^ and meta-analyses^4^ have shown that high levels of psychosocial risk factors are associated with an increased risk of IHD. Anxiety^5^ and depression 6 are among the most prominent psychological risk factors for incident ACS and adverse outcomes in patients with IHD.^7^ However, depression has been extensively studied^8^ and has an official recommendation in clinical cardiology guidelines.^9^,^10^ Anxiety has received less attention, but multiple studies show its importance in predicting higher rates of cardiac risk factors and events.^11^,^12^ Findings on anxiety and patients with suspected myocardial ischemia have been mixed,^13^,^14^ warranting further investigation. The population attributable risk of psychological factors for incident CAD events is found to be higher for women than men because women report higher levels of distress-related psychological factors than men.^15^,^16^ However, the relative risk of incident CAD events related to psychological factors does not markedly differ between women and men.^4^,^17^ Specifically, the prevalence of anxiety is higher among women compared to men.^18^ In addition, especially younger women in the cardiac patient population (compared to the general population) have significantly more anxiety-related problems than older women and men.^19^ Although evidence indicates that younger age may be associated with different pathophysiological processes in women with IHD,^20^,^21^ it is unclear whether the association of anxiety with inducibility of ischemia is age dependent.

Epidemiological data indicate that women younger than 65 years of age display increasing rates of acute coronary syndromes (ACS) relative to men in the same age range.^22^,^23^ Hospitalization and 30-day mortality rates are also increasing in women younger than 55 years of age compared to men.^24^ Elevated hemodynamic reactivity in younger women may partially explain the relatively high prevalence of myocardial ischemia in response to adenosine administration during cardiac stress testing.^21^ For example, baseline heart rate and baseline systolic blood pressure (SBP) were significantly higher in women compared with men during adenosine cardiac stress testing, and SBP significantly increased during stress testing for women but not for men.^21^ The gender differences in IHD incidence and progression as well as inducibility of ischemia indicate that further investigations are needed on the interplay between gender, age, and cardiovascular risk factors.

High levels of trait anxiety are associated with higher rates of ischemia in women without a history of CAD compared to women with lower levels of trait anxiety.^25^ Another study showed that, among patients with non-obstructive CAD, the risk of developing ischemia during cardiac stress testing was higher among women with high levels of trait anxiety, but not state anxiety.^26^ It has also been documented that trait anxiety is associated with more frequent and severe cardiac symptoms.^27^ The association between anxiety and ischemia may be accounted for in part by higher hemodynamic responses during cardiac stress testing. Anxiety is generally related to increased sympathetic nervous system activity.^28^ In addition, elevated levels of anxiety have been associated with higher sympathetic nervous system activity in postmenopausal women.^29^ These data indicate that there may be an interplay between gender, age, anxiety, and hemodynamic responsiveness as related to inducible myocardial ischemia during cardiac stress testing.

The present study examines whether anxiety plays a role in the gender differences in hemodynamic reactivity and inducible ischemia during cardiac stress testing. Given that evidence suggests that these associations seem to be stronger in young women, we will also explore age-stratified analyses. Specifically, it was hypothesized that: (1) women will report higher levels of anxiety than men; (2) high levels of anxiety in patients undergoing cardiac stress testing will be associated with elevated hemodynamic reactivity in women, and less so in men; (3) high levels of anxiety will be associated with the presence and severity of inducible ischemia during cardiac stress testing in women, but less in men. Furthermore, we expect that these gender-specific associations primarily occur among female patients of younger age (≤ 65 years) versus older age (> 65 years).

## Methods

### Patients

The study sample consisted of 291 patients who underwent a pharmacological or exercise stress/rest myocardial perfusion imaging single-photon emission computed tomography (MPI-SPECT, henceforth referred to as MPI) protocol with adenosine (n = 203) (140 mcg^−1^ kg^−1^ min^−1^ for 5 minutes) or exercise (n = 88) (i.e., bicycling to maximum exertion using the modified Bruce protocol) between January 2017 and December 2018 at Institute Verbeeten Tilburg, the Netherlands. Inclusion criteria were: (1) referral for the MPI protocol with adenosine or exercise; (2) ability to fill out questionnaires; and (3) sufficient knowledge of the Dutch language. There were no exclusion criteria. Reason for referral were categorized into ‘examination of new or worsening symptoms (no previously known CAD)’ (N = 174), ‘examination of known CAD (returning symptoms)’ (N = 114), or ‘preoperative cardiovascular risk assessment’ (N = 3). All patients underwent the protocol as described below with MPI images obtained at rest and following cardiac stress testing.

In order to obtain state anxiety measures, patients’ facial expressions were video-recorded using a webcam (Logitech C920-HD Pro) attached to the stationary exercise bicycle, for both adenosine and exercise stress testing protocols (GE Healthcare, Ergometer ebike comfort 162202, Freiburg, Germany), as further described below. During cardiac stress testing (both protocols), patients were asked whether they experienced anginal chest pain symptoms (anginal chest pain present/absent) similar to their “typical” cardiac symptoms.

Sociodemographic and psychosocial data were collected before and after cardiac stress testing. Information on cardiovascular risk factors (hypertension, hypercholesterolemia, familial risk, and smoking), medical history, and medication was obtained from electronic patient records. In the present study, we refer to differences between women and men as gender rather than sex differences, since in human study participants the contributions of social, environmental, cultural, and behavioral factors and choices cannot be excluded as factors influencing the research findings.^30^ The study was approved by the regional Medical Ethics Committee (METC Brabant, Protocol number: NL56707.028.16), and all patients provided written informed consent.

### Myocardial Perfusion Imaging (Rest-Stress Protocol)

All patients underwent a two-day protocol with the first day allocated to the at-rest imaging and the second day to the stress (adenosine or exercise) imaging. Patients were instructed to refrain from consuming caffeine-containing beverages for 24 hours before each protocol day. The first day consisted of ^99m^Tc-tetrofosmin injection (dosage: 370 MBq), and a rest period of 45 minutes followed by myocardial perfusion imaging. The second day, patients performed cardiac stress testing, either pharmacologically by intravenous adenosine injection (140 mcg^−1^ kg^−1^ min^−1^ for 5 minutes) or by exercise (i.e., bicycling to maximum exertion using the modified Bruce protocol). As per standard clinical protocol, the pharmacological adenosine cardiac stress testing also involved mild-intensity cycling to limit adenosine side-effects and reduce extracardiac activity.^31^ For the adenosine protocol, ^99m^Tc-tetrofosmin injection was at 2 minutes after adenosine administration. During the exercise protocol, the injection was at peak exercise, at 85% of the maximum heart rate (0.85*(220-patient age)).

Image acquisition was performed using a hybrid dual-headed gated IQ SPECT/CT system (Symbia T, Siemens Medical Solutions AG) equipped with multifocal collimators (SmartzoomTM) of 128 × 128 matrix size and zoom factor of 1. Acquired data were then reconstructed using an iterative reconstruction. Attenuation correction was applied using a patient-dedicated low-dose CT-derived mu map. A symmetric 15% window was centered at 140 keV, with a three-lead electrocardiographic monitoring. Perfusion images were inspected by qualified staff before interpretation by experienced observers.

### Image Analysis and Hemodynamic Reactivity Parameters

Bull’s eye generation, and visual analysis using a 17-segmental model,^32^ was performed by two experienced observers. Semi-quantitative analyses of perfusion were performed with QPS software from Cedars-Sinai Medical Center. The interpretation of the scan was assessed both semiquantitatively and by visual analysis, as is recommended by the American Society of Nuclear Cardiology (ASNC).^33^ Both AC and no-AC images were reviewed during interpretation. Perfusion was graded on a 0-4 scale: 0 = normal, 1 = equivocal, 2 = moderate, 3 = severe perfusion defect, and 4 = very severe perfusion defect. Quantitative summed rest scores (SRS), summed stress scores (SSS), and summed difference scores (SDS) were tabulated. Myocardial ischemia was semiquantitatively defined as SDS ≥ 2.^34^ Pixel-wise total perfusion deficit (TPD) was used as an automated perfusion deficit parameter and TPD ≥ 3% was considered abnormal and indicative of a perfusion defect.^35^

#### Hemodynamic Reactivity

Baseline and peak heart rate (HR) and blood pressure (BP) were recorded in beats per minute (bpm) and mmHg, respectively. Peak HR was defined as the HR at 2 minutes after adenosine injection and maximum heart rate during the exercise protocol. Peak BP was recorded at 2 minutes after adenosine injection and at peak exertion during the exercise protocol. HR response for both protocols was defined as the % heart rate reserve (HRR, (peak HR − baseline HR)/baseline HR)*100). Left ventricular ejection fraction (LVEF) was determined, and also end diastolic volume (EDV) and end systolic volume (ESV) were calculated.

### Trait Anxiety Measure

The generalized anxiety disorder (GAD)-7 scale is a clinical screening measure for assessing generalized anxiety disorder. The GAD-7 consists of seven items that are answered on a four-point scale from 0 (“not at all”) to 3 (“nearly every day”). The GAD-7 has been validated in the general population,^36^ yielding a high internal consistency (Cronbach’s alpha = .89). For analyses, the continuous score of the GAD-7 was used, with a higher score indicating more anxiety symptoms. A cut-off score of 10 is also used indicating patients with ‘low anxiety’ (GAD-7 ≤ 10) and ‘high anxiety’ (GAD-7 > 10).

### State Anxiety Measure

#### Video-Recording

State anxiety measures were based on analyzed video-recordings during cardiac stress testing for both the adenosine and exercise protocol. For the analyses of anxiety expressions, the software package FaceReader 7.0^37^ was used to analyze the video-recordings made during cardiac stress testing. Although this is a relatively novel method, it has been validated and used in multiple research settings.^38^-^40^ After taking a seat on the exercise bicycle (both adenosine and exercise), the video-recordings started with ‘baseline’, approximately 1 minute before cardiac stress testing actually began, and served as the baseline measure of facial expression of anxiety. The three other time blocks during cardiac stress testing were ‘start cardiac stress testing’ when patients started exertion, ‘maximal cardiac stress testing’, when heart rate was at peak level (or at 2 minutes after adenosine injection), and ‘recovery’, when patients stopped exertion and slowly recovered.

#### FaceReader Analysis of Video-Recordings

FaceReader first synthesizes an artificial face model, which describes the location of over 500 keypoints in the face and the facial texture of the area entangled by these points. Then, based on this, classification of the facial expressions is done by an artificial neural network.^41^ Based on over 10.000 images, the network was trained to classify the six universal emotions: anxiety, sadness, anger, surprise, happiness, and disgust.^42^ Finally, scores of intensity of facial expressions are computed on a continuous scale from 0 to 1, yielding the percentage (0%-100%) of facial expression of anxiety. In addition to the percentage of facial expression of anxiety, a dichotomous measure (median split) of facial expression of anxiety is used in statistical analyses.

### Statistical Analysis

Data are presented as means ± standard deviation (SD) for continuous variables and frequencies and percentages for categorical variables, stratified by gender. Group comparisons for categorical variables were examined using *χ*^2^ tests and continuous variables using independent t-tests, for the adenosine and exercise protocols separately. Associations of ischemia with trait and state anxiety and the interaction with gender and age were examined using a two-way ANOVA with a gender by (trait and state) anxiety interaction term, stratified for two age groups: ≤ 65 and ≥ 65 years of age. Residual analysis was performed to test for the assumptions of the two-way ANOVA. Outliers were assessed by inspection of a boxplot, and homogeneity of variances was assessed by Levene’s test. SRS, SSS, and SDS were square root transformed to account for outliers. The primary outcome measure is ischemia, semi-quantitative operationalized as SDS, and SSS, inferring diagnostic value.^43^ Hemodynamic measures included HR, HRR, SBP, DBP, and LVEF. Statistical analyses were performed using SPSS version 24.0 (SPSS Inc., Chicago, Illinois), two-sided *P* values are reported and statistical significance was set at *P* < 0.05.

## Results

### Patient Characteristics

Table 1 displays the patient characteristics. The mean age of the sample was 66.8 ± 8.7 years. The mean age for women was slightly lower (65.8 ± 8.5) than for men (67.7 ± 8.8; *P *= .071). Men more often had a previous MI (16% vs 5%, *P* = .006), PCI (34% vs 18%, *P* = .002), and CABG (22% vs 5%, *P* < .001) than women. Women more often had no history of previously known CAD compared to men (72% vs 50%, *P =* .001).

**Table 1:** Patient characteristics stratified by gender

Baseline characteristic	Women (N = 130)	Men (N = 161)	*P* value
Age (years)	65.8 ± 8.5	67.7 ± 8.8	.071
BMI (kg/m^2^)	28.9 ± 6.7	27.9 ± 4.5	.155
Smoking (yes)	16 (13%)	25 (16%)	.451
Physical activity (sufficiently active)*	106 (84%)	135 (86%)	.662
Hypertension	56 (49%)	72 (50%)	.845
Hypercholesterolemia	56 (51%)	65 (47%)	.471
Diabetes Mellitus	37 (23%)	28 (22%)	.769
Familial risk (first degree family member)	**82 (65%)**	**84 (53%)**	**.043**
Previous MI	**7 (5%)**	**25 (16%)**	**.006**
Previous PCI	**23 (18%)**	**55 (34%)**	**.002**
Previous CABG	**6 (5%)**	**35 (22%)**	**< .001**
Reasons for referral			
Examination of new or worsening symptoms (no previously known CAD)	**93 (72%)**	**81 (50%)**	**.001**
Examination of known CAD (recurring symptoms)	**37 (29%)**	**77 (48%)**	
Preoperative cardiovascular risk assessment	**0 (0%)**	**3 (2%)**	
Medication			
Anticoagulants	**83 (64%)**	**128 (80%)**	**.003**
ACE-ARB inhibitors	*53 (41*%*)*	*90 (56*%*)*	*.010*
Beta-blocker	*61 (47*%*)*	*92 (57*%*)*	*.083*
Calcium inhibitors	**24 (19%)**	**54 (34%)**	**.004**
Diuretics	34 (26%)	34 (21%)	.313
Statins	**69 (53%)**	**113 (70%)**	**.003**
Nitrates	37 (29%)	58 (36%)	.171
Diabetes medication	31 (19%)	24 (19%)	.864
Antidepressants	**18 (14%)**	**8 (5%)**	**.008**
Anxiety			
Trait anxiety (GAD-7 mean score)	5.48 ± 5.2	5.49 ± 5.6	.982
Trait anxiety (GAD-7 > 10)	23 (18%)	26 (18%)	.883

Data presented as mean ± standard deviation or number (%)

Bold values indicate *P* < .05

Italic values indicate *P* < .10

* Based on 30 minutes of daily moderate activity

*BMI*, Body Mass Index; *MI*, Myocardial Infarction; *PCI*, percutaneous coronary intervention; *CABG*, coronary artery bypass grafting; *GAD*, Generalized Anxiety Disorder

### Gender Differences in Myocardial Ischemia

Myocardial ischemia was present in 107/291 (37%) of the patients. Overall, women less often displayed ischemia than men; 28% vs 39% (*X*^2^ = 6.98, *P *= .008), and women had lower mean SRS (1.16 ± 2.83 vs 2.59 ± 5.0, *P *= .004), SSS (2.52 ± 4.74 vs 4.12 ± 6.16, *P *= .016), and TPD (2.09 ± 3.27 vs 3.18 ± 4.17, *P* = .016) values than men, and a similar but non-significant difference was found for SDS (1.45 ± 2.46 vs 1.84 ± 3.13, *P* = .253). We also explored the interaction between gender and age as related to ischemia, but no significant interactions between gender and age were found with regard to SRS, SSS, SDS and TPD values (all *P* > .753).

Table 2 shows the analyses per cardiac stress testing protocol (adenosine and bicycle exercise). Ischemia occurred in 40% of the 203 patients undergoing the adenosine protocol and 28% in the 88 patients undergoing the bicycle exercise protocol. During the adenosine protocol, men displayed slightly more ischemia then women (46% vs 34%) but this difference was not significant (*P* = .077) (Table 2). During the exercise protocol, ischemia was more prevalent among men than women (38% vs 16%, *P* = .022).

**Table 2:** MPI acquisition and hemodynamics at rest and during stress stratified for adenosine and exercise protocol

MPI variables and hemodynamics	Adenosine protocol (n = 203)			Exercise protocol (n = 88)		
	Women (n = 92)	Men (n = 111)	*P* value	Women (n = 38)	Men (n = 50)	*P* value
SDS score	1.8 ± 2.7	2.0 ± 3.4	.719	**0.6 ± 1.3**	**1. 5 ± 2.3**	**.013**
SSS score	**3.1 ± 5.2**	**4.9 ± 6.9**	**.046**	**1.0 ± 3.0**	**2.5 ± 3.5**	**.037**
Ischemia based on SDS score ≥ 2	*31 (34%)*	*51 (46%)*	*.077*	**6 (16%)**	**19 (38%)**	**.022**
TPD	2.6 ± 3.6	3.6 ± 4.5	.101	**0.8 ± 1.6**	**2.3 ± 3.1**	**.003**
Typical anginal chest pain present	*43 (47%)*	*38 (34%)*	*.070*	8 (21%)	8 (16%)	.543
LVEF						
LVEF rest (%)	**64.2 ± 11.8**	**53.7 ± 11.6**	**< .001**	**68.2 ± 9.7**	**55.5 ± 10.2**	**< .001**
LVEF stress (%)	**62.4 ± 11.1**	**50.5 ± 11.2**	**< .001**	**68.4 ± 9.0**	**57.2 ± 11.3**	**< .001**
EDV rest (ml)	**73.1 ± 35.4**	**105.8 ± 37.9**	**< .001**	**64.2 ± 15.5**	**102.3 ± 27.6**	**< .001**
ESV rest (ml)	**30.1 ± 27.9**	**52.0 ± 33.2**	**< .001**	**21.5 ± 11.6**	**47.2 ± 19.9**	**< .001**
EDV post-stress (ml)	**74.6 ± 24.9**	**113.5 ± 47.1**	**< .001**	**63.1 ± 18.1**	**98.5 ± 29.1**	**< .001**
ESV post-stress (ml)	**30.2 ± 18.7**	**59.7 ± 41.2**	**< .001**	**21.4 ± 11.6**	**44.3 ± 20.8**	**< .001**
Diastolic blood pressure (DBP)						
Baseline (mmHg)	79.3 ± 13.0	78.7 ± 12.9	.755	82.0 ± 8.7	79.4 ± 12.9	.281
Peak (mmHg)	73.6 ± 14.8	76.2 ± 12.3	.183	84.4 ± 21.6	90.4 ± 18.5	.163
Systolic blood pressure (SBP)						
Baseline (mmHg)	*133.3 ± 26.2*	*127.3 ± 23.1*	*.091*	131.7 ± 21.6	128.5 ± 15.9	.426
Peak (mmHg)	141.1 ± 29.9	139.86 ± 22.7	.729	*165.7 ± 33.0*	*178.3 ± 29.2*	*.062*
Heart rate						
Baseline (bpm)	72.1 ± 12.1	70.0 ± 11.7	.208	**77.9 ± 12.7**	**71.7 ± 14.5**	**.039**
Peak (bpm)	**100.4 ± 16.8**	**90.8 ± 18.4**	**< .001**	128.5 ± 24.0	135.8 ± 20.1	.122
HRR (%)	**40.9 ± 23.3**	**30.7 ± 22.7**	**.002**	**67.3 ± 34.5**	**96.3 ± 48.9**	**.002**
State anxiety						
Rest	2.8 ± 3.0	2.8 ± 2.6	.939	2.5 ± 0.4	3.3 ± 2.6	.165
Stress	3.1 ± 3.1	3.0 ± 3.8	.923	2.6 ± 2.2	3.8 ± 5.9	.192

Data presented as mean ± standard deviation or number (%)

Bold values indicate *P* < .05

Italic values indicate *P* < .10

*SDS*, summed difference score; *SSS*, summed stress score; *TPD*, total perfusion deficit; *LVEF*, left ventricular ejection fraction; *EDV*, end diastolic volume; *ESV*, end systolic volume; *DBP*, diastolic blood pressure; *SBP*, systolic blood pressure; *HRR*, heart rate response

During the adenosine protocol, SSS was higher for men compared to women (4.9 ± 6.9 vs 3.1 ± 5.2, *P *= .046). TPD was higher for men only during the exercise protocol (0.8 ± 1.6 vs 2.3 ± 3.1, *P* = .003). No differences were found for typical anginal chest pain between men and women for both protocols.

### Gender Differences in Trait and State Anxiety

No significant gender differences were found for the presence of trait anxiety (18% vs 18%, *P* = .883) with similar total scores (women 5.48 ± 5.2 vs men 5.49 ± 5.6; *P* = .982). When comparing the sample by age, no significant differences were found for the presence of trait anxiety (23% (younger patients) vs 14% (older patients), *P* = .058). The interaction between gender and age as related to trait anxiety was also not significant (*F*(1, 270) = .633, *P* = .427, partial *η*^2^ = .002)

Measures of state anxiety based on facial expression revealed that no gender differences were found for presence of state anxiety for all time blocks (baseline: 44% vs 38%, *P* = .270; start cardiac stress testing: 38% vs 35%, *P* = .660; max cardiac stress testing: 47% vs 40%, *P* = .260; recovery: 45% vs 38%, *P* = .189). Mean scores for each time block were also similar for men and women (baseline: 2.94 vs 2.74, *P* = .552; start cardiac stress testing: 2.52 vs 2.60, *P* = .796; max cardiac stress testing: 3.26 vs 2.90, *P* = .449; recovery: 3.77 vs 3.33, *P* = .267). The interaction between gender and age as related to state anxiety was not significant for all time blocks (all *P* > .252).

### Gender and Age Differences in Hemodynamic Reactivity During Cardiac Stress Testing

Women had a higher heart rate after 2 minutes of adenosine injection (100.4 ± 16.8 bpm vs 90.8 ± 18.4 bpm, *P* < .001) and HRR (40.9 ± 23.3 vs 30.7 ± 22.7, *P *= .002) than men. Baseline heart rate was higher for women prior to the exercise protocol (77.9 ± 12.7 bpm vs 71.7 ± 14.5 bpm, *P *= .039) and no gender differences for peak heart rate or HRR during exercise were found. No significant differences in blood pressure during rest or peak were found between men and women for both protocols (Table 2).

As expected, LVEF values were higher in women than men during both rest (64.2% ± 11.8% vs 53.7% ± 11.6%, *P* < .001) and cardiac stress testing (62.4% ± 11.1% vs 50.5% ± 11.2%, *P* < .001). LVEF decreased during the adenosine protocol for women (64.9% ± 10.9% to 62.5% ± 10.8%, *P* = .002) and men (53.3% ± 11.6% to 51.1% ± 10.9%, *P* = < .001). During the exercise protocol, LVEF significantly increased for men (55.5% ± 10.3% to 57.6% ± 11.1%, *P *= .018), but not for women (68.2% ± 9.7% to 68.4% ± 9.0%, *P* = .835).

When examining age groups, patients under 65 years in the adenosine protocol had a significantly higher peak heart rate (at 2 minutes after adenosine injection) than patients older than 65 years (100.3 ± 16.0 vs 92.4 ± 18.8 bpm, *P* = .002). Patients older than 65 years had a higher rest systolic blood pressure than patients younger than 65 years (133.9 ± 25.2 vs 122.5 ± 22.0 mmHg, *P* = .001). In the exercise protocol, patients under 65 years had a significantly higher peak heart rate than patients older than 65 years (140.4 ± 19.4 vs 125.1 ± 22.0 bpm, *P* = .001). No significant differences in blood pressure were found between age groups. No significant differences were found in LVEF for both protocols.

### Trait Anxiety and Hemodynamic Reactivity

Patients with high trait anxiety in the adenosine protocol (n = 41) had significantly higher baseline heart rate (74.0 ± 10.0 vs 70.1 ± 12.1 bpm, *P* = .038) and peak heart rate (102.9 ± 17.3 vs 93.4 ± 18.2 bpm, *P* = .003) than patients with low anxiety (n = 152, reported numbers smaller due to 10 missing values for GAD-7). Patients with high trait anxiety (n = 8) in the exercise protocol had a significantly lower baseline LVEF (53.4% ± 7.9% vs 62.3 ± 11.4%, *P* = .025) than patients with low trait anxiety (n = 73, reported numbers smaller due to seven missing values for GAD-7).

### State Anxiety and Hemodynamic Reactivity

During start cardiac stress testing, patients with high state anxiety during the adenosine protocol (n = 76) had a lower systolic blood pressure than patients with low state anxiety during cardiac stress testing (n = 127) (125.5 ± 20.5 vs 132.8 ± 26.7 mmHg, *P* = .032). No other differences in hemodynamic reactivity were found for patients undergoing the adenosine protocol. In the exercise protocol, patients with high state anxiety during recovery (n = 43) had a higher peak LVEF than patients with low state anxiety (n = 39) (64.7% ± 11% vs 59.2% ± 11.9%, *P* = .030). No other differences were found in hemodynamic reactivity for patients in the exercise protocol.

### Trait Anxiety and Ischemia

There was no significant overall association between trait anxiety with inducibility of ischemia, SSS, SDS, or TPD values (all *P* > .221 and partial *η*^2^ < .006). There were also no gender-by-trait anxiety interactions for these measures (*P* values > .217; partial *η*^2^ < .003).

**Figure 1:**
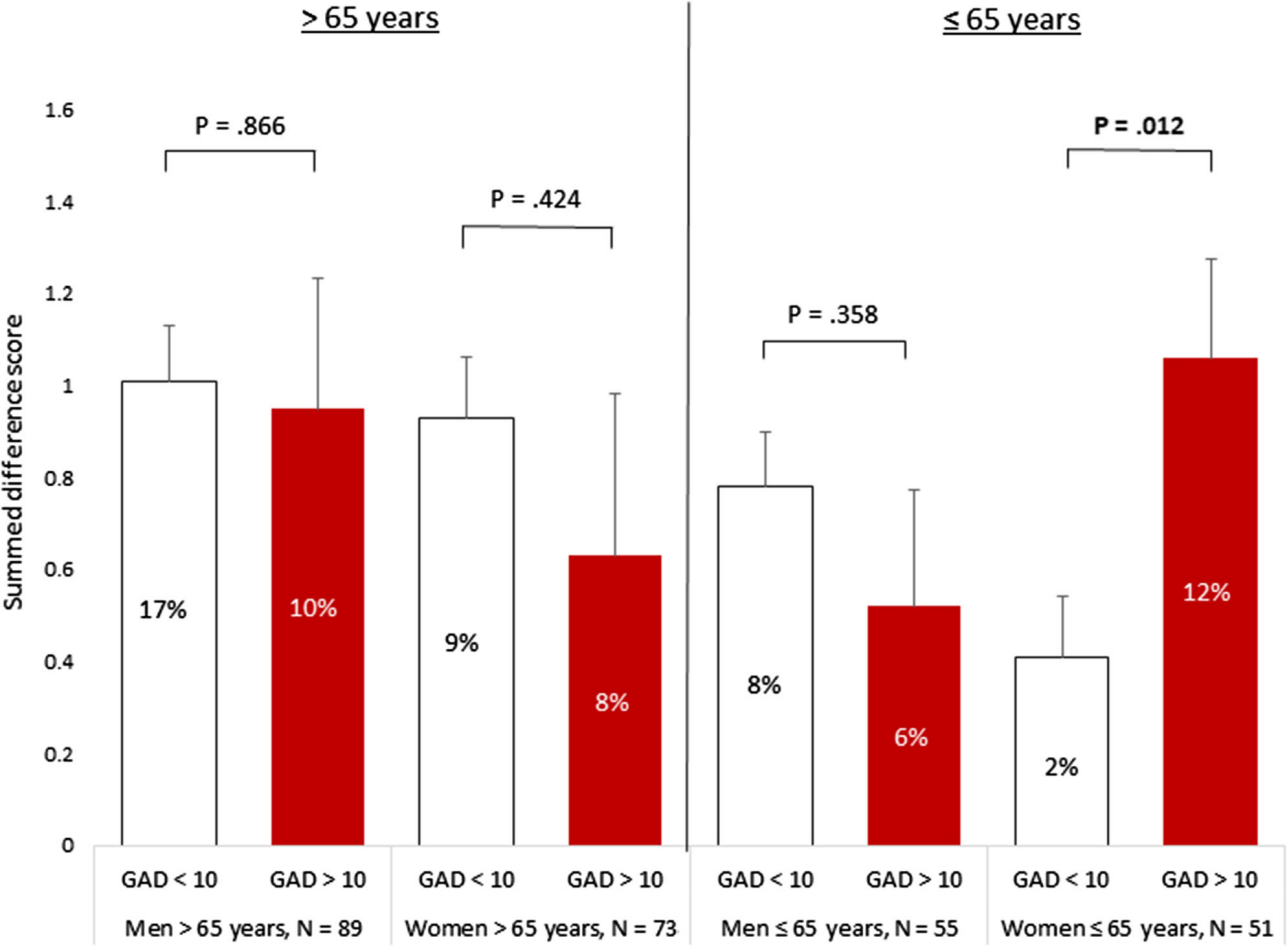
Summed Difference Score (SDS, square root transformed) stratified for trait anxiety (high/low), gender and age groups. The presence of ischemia is presented in the bar graphs as % per subgroup

**Figure 2:**
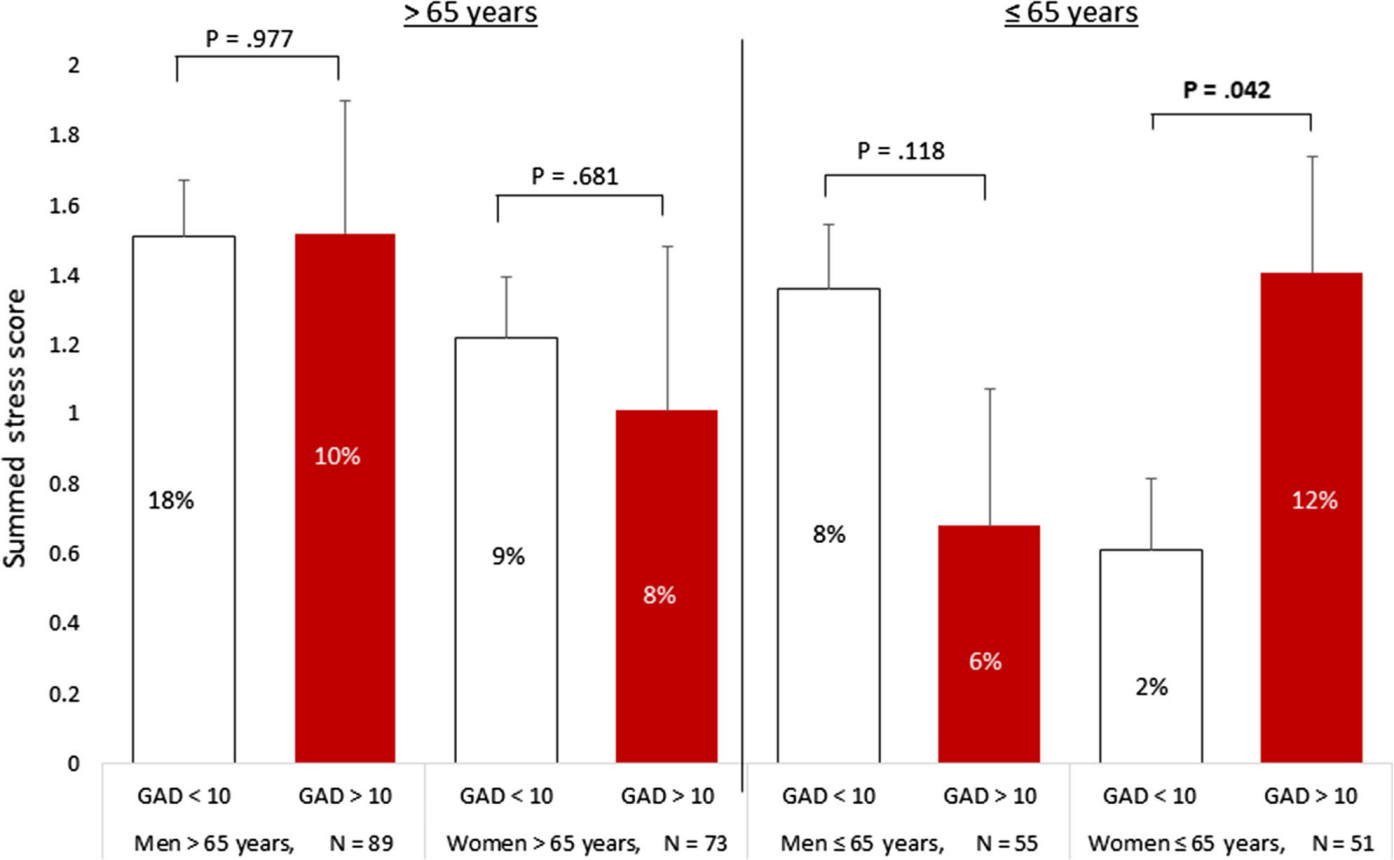
Summed Stress Score (SSS, square root transformed) stratified for trait anxiety (high/low), gender and age groups. The presence of ischemia presented in the bar graphs as % per subgroup

However, as shown in Figures 1 and 2, significant interaction effects were found between gender and trait anxiety when stratifying by age. For individuals ≤ 65 years, the gender x trait anxiety interactions were significant for SDS (*F*(1, 4) = 5.73, *P* = .019, partial *η*^2^ = .053), SSS (*F*(1,10) = 6.50, *P* = .012, partial *η*^2^ = .060), and TPD (*F*(1, 26) = 5.71, *P* = .019, partial *η*^2^ = .053), for patients under 65 years of age. For women with high trait anxiety the mean SDS was 0.65 (95% CI: 0.14-1.15) higher than in women with low trait anxiety. Similarly, the mean SSS was 0.80 (95% CI: 0.03-1.57) higher than women with low anxiety scores, and the mean TPD was 1.91 (95% CI: 0.59-3.23) higher in women with high vs low trait anxiety. These associations with trait anxiety were not observed in men (Figures 1 and 2).

For patients > 65 years, a significant interaction effect was found for gender with trait anxiety for TPD only (*F*(1, 80) = 4.05, *P* = .046, partial *η*^2^ = .025). For patients with high trait anxiety, the mean TPD was 4.71 (95% CI 0.96-8.46) higher for men than for women, whereas other subgroup comparisons were not significant.

### State Anxiety and Ischemia

A two-way ANOVA showed that the interaction effect between state anxiety and gender for SSS, SDS, and TPD was not statistically significant for either of the four time blocks (baseline, start cardiac stress testing, max cardiac stress testing, and recovery) during cardiac stress testing (all *P* values ≥ .071 and partial *η*^2^ < .023) for both patients under and over 65 years.

## Discussion

The present study examines whether anxiety plays a role in the gender differences in hemodynamic reactivity and inducible ischemia during cardiac stress testing. The first hypothesis was not confirmed, as men and women did not differ on overall trait and state anxiety. The second hypothesis was confirmed, since women had higher heart rate responses to cardiac stress testing when undergoing the adenosine protocol and, overall, a higher heart rate response was associated with high trait anxiety. For the third hypotheses, we found that measures of ischemia (SDS and SSS), albeit low in each subgroup, were significantly higher in women with high trait anxiety, but only in patients younger than 65 years of age.

Gender differences during cardiac stress testing were examined for both the adenosine and exercise protocol. For the adenosine protocol, the peak heart rate and HRR were significantly higher in women compared to men. This difference was not observed during the exercise protocol, in which peak heart rate was higher for men and no gender differences in HRR were found. Consistent with these results, previous studies determined gender and age as independent factors associated with HR responses.^44^,^45^ Mechanisms for this gender difference are not yet fully clear, but research suggests that women exhibit higher parasympathetic stimulation of the heart, which is ultimately protective during periods of increased cardiac stress.^46^ However, no marked gender differences were found for SBP and DBP during baseline and peak for both protocols. Consistent with earlier studies, SBP was slightly blunted during adenosine in women, but not in men.^47^ A significantly higher LVEF was observed in women compared to men for both protocols which is consistent with previous findings.^48^ Reduced LVEF has been associated with inducibility of myocardial ischemia,^49^ which may partially explain the overall higher prevalence of ischemia in men than women.

For patients younger than 65 years, a significant interaction effect was found between trait anxiety, but not state anxiety, and gender. Being female, younger than 65 years, and having high trait anxiety was associated with a significantly higher SSS, SDS, and TPD compared to men. This finding was not observed in patients of 65 years and older. The observed state versus trait anxiety contrast in association with ischemia is consistent with earlier findings.^26^,^50^ These two studies, however, were conducted with a healthy elderly (male) patient sample and a cardiac syndrome X patient sample, respectively, suggesting that trait anxiety is a recurring significant factor in both patients with and without cardiac history and studies with different cardiac outcomes. In the study by Paine and colleagues,^25^ trait anxiety in women with no CAD history was significantly related to increased ischemia extent compared to women without anxiety, which is comparable to the findings by Vermeltfoort and colleagues,^26^ but in the study of Paine, state anxiety was not examined. In the present study, both trait and state anxiety were examined in relation to ischemia and groups were stratified for age, as is done in the study by Gebhard and colleagues,^21^ which concludes that younger women with ischemia have a stronger hemodynamic response to adenosine stress testing than men, a finding that was partly confirmed in our study when comparing women to men.

Consequently, when examining previous studies, our finding that trait anxiety is related to myocardial ischemia in younger women could possibly be attributed to increased sympathetic nervous system activity inherent to anxiety.^51^ Increased sympathetic nervous system activity could result in an increased presence of ischemia through increases in heart rate and vasoconstriction.^52^ However, increased sympathetic nervous system activity is beyond the scope of the present study and requires additional research. In addition, it is unclear whether this rationale would be more applicable to trait anxiety or state anxiety.

The present study had some strengths and limitations that should be taken into consideration. Limitations include the relatively small patient sample and the cross-sectional design, making discussions on causality only speculative. The incorporation of a validated questionnaire to measure trait anxiety can be considered a strength of this study. A unique aspect is the measurement of state anxiety with facial expression recognition software during cardiac stress testing. Because of the transient nature of cardiac stress testing, it should be linked with a transient measure of facial expressions of emotions to optimally reflect the dynamic association between emotions and ischemia.^53^ The low intensity of detected state anxiety during cardiac stress testing for men and women (under 10%), could account for the non-significant reported findings for state anxiety in this study.

When evaluating the gender differences in patient characteristics, it was observed that men more often had an elaborate cardiac history compared to women, which is consistent with previous studies.^54^ Reasons accounting for this difference are discussed with increasing attention for psychological factors.^55^,^56^ Furthermore, no significant differences in typical anginal chest pain during cardiac stress testing were found for men and women in both protocols. This study also confirms the trait and state anxiety contrast reported in previous studies and suggests that high trait anxiety is an important factor to take into account in women with myocardial ischemia. Larger studies with more sociodemographic variation in the patient groups are needed to further clarify the risk of anxiety in patients with myocardial ischemia.

To conclude, this study shows that female patients younger than 65 years of age with high trait anxiety have increased SDS, SSS, and TPD following cardiac stress testing compared with men and patients older than 65 years. Thus, the implications of psychological constructs on cardiac health are intertwined with gender and age. This interplay is worthy of further investigation and larger observational and intervention studies in patients who are at high risk of myocardial ischemia and adverse long-term cardiovascular prognosis.

## New Knowledge Gained

Our data indicate that SDS and SSS were significantly higher in women younger than 65 years with high trait anxiety. This interaction was not found in men and women over 65 years. State anxiety is not significantly associated with myocardial ischemia.

## Electronic supplementary material

Below is the link to the electronic supplementary material.

Electronic supplementary material 1 (PPTX 200 kb)

Electronic supplementary material 2 (M4A 3129 kb)

## Abbreviations

CAD: Coronary artery disease
HRR: Heart rate reserve
LVEF: Left ventricular ejection fraction
IHD: Ischemic heart disease
MPI: Myocardial perfusion imaging
SDS: Summed difference score
SRS: Summed rest score
SSS: Summed stress score
TPD: Total perfusion deficit
