# Supplementary material for: Anxiety and hemodynamic reactivity during cardiac stress testing: The role of gender and age in myocardial ischemia
Source: J Nucl Cardiol. 2020 Feb 28;28(6):2581–92. doi: 10.1007/s12350-020-02079-3 (PMC8709819; doi:10.1007/s12350-020-02079-3)
Supplement: Supplementary file 1 — Electronic supplementary material 1 (PPTX 200 kb) [file 12350_2020_2079_MOESM1_ESM.pptx]

## Slide 1
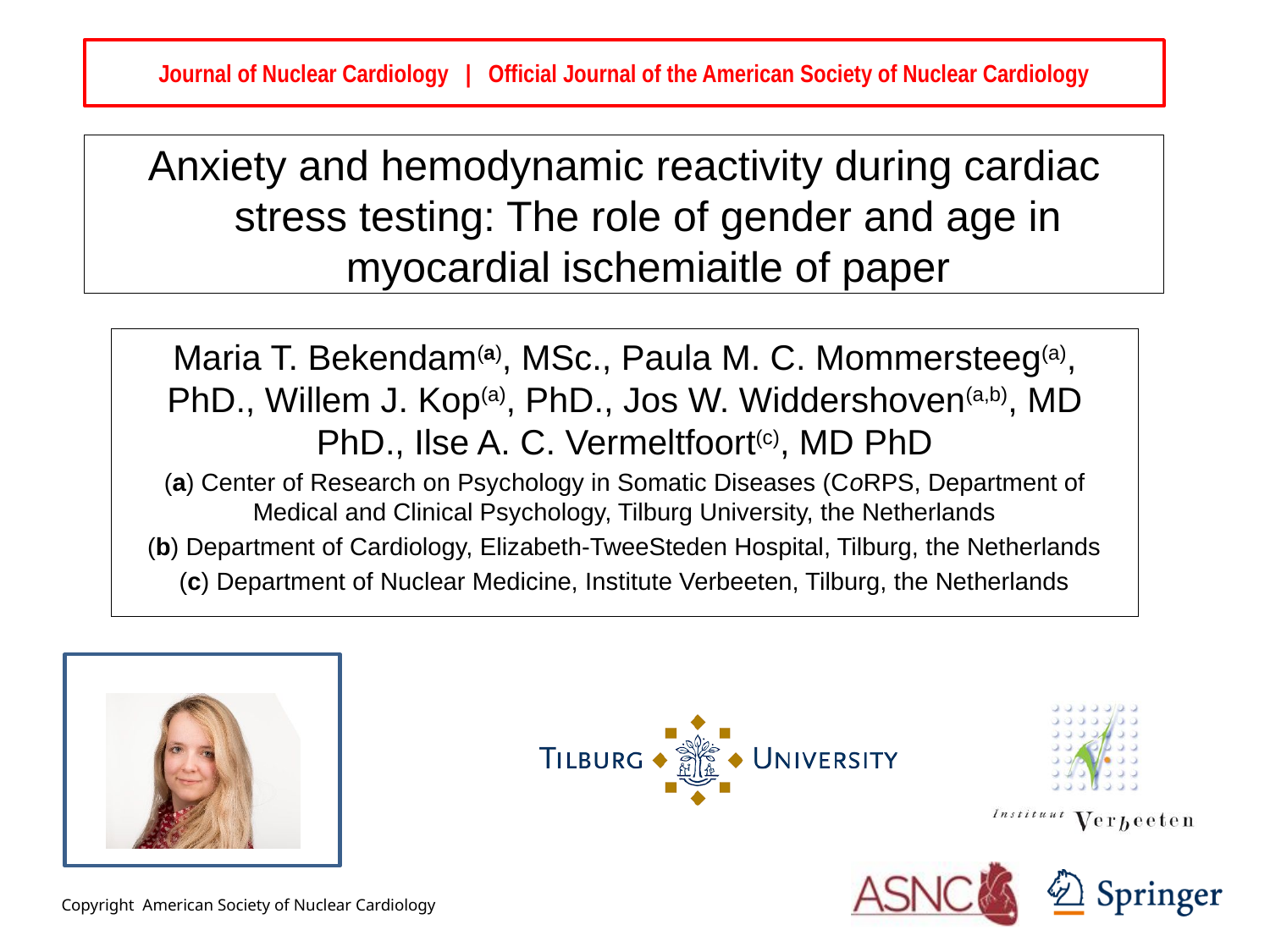

Journal of Nuclear Cardiology | Official Journal of the American Society of Nuclear Cardiology
# Anxiety and hemodynamic reactivity during cardiac stress testing: The role of gender and age in myocardial ischemiaitle of paper
Maria T. Bekendam(a), MSc., Paula M. C. Mommersteeg(a), PhD., Willem J. Kop(a), PhD., Jos W. Widdershoven(a,b), MD PhD., Ilse A. C. Vermeltfoort(c), MD PhD
(a) Center of Research on Psychology in Somatic Diseases (CoRPS, Department of Medical and Clinical Psychology, Tilburg University, the Netherlands
(b) Department of Cardiology, Elizabeth-TweeSteden Hospital, Tilburg, the Netherlands
(c) Department of Nuclear Medicine, Institute Verbeeten, Tilburg, the Netherlands
Head shot of author
required
Copyright American Society of Nuclear Cardiology

## Slide 2
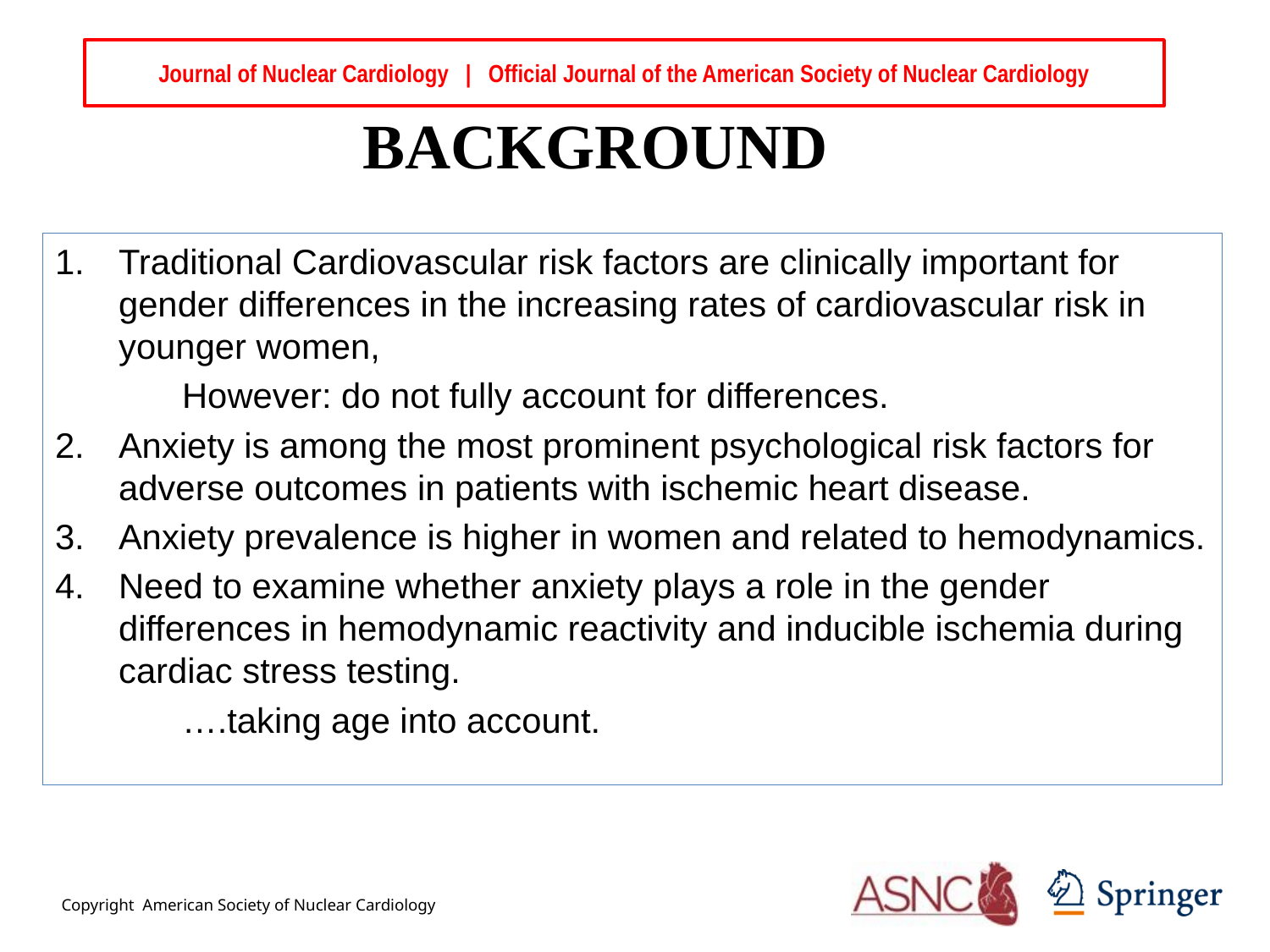

Journal of Nuclear Cardiology | Official Journal of the American Society of Nuclear Cardiology
# BACKGROUND
Traditional Cardiovascular risk factors are clinically important for gender differences in the increasing rates of cardiovascular risk in younger women,
	However: do not fully account for differences.
Anxiety is among the most prominent psychological risk factors for adverse outcomes in patients with ischemic heart disease.
Anxiety prevalence is higher in women and related to hemodynamics.
Need to examine whether anxiety plays a role in the gender differences in hemodynamic reactivity and inducible ischemia during cardiac stress testing.
	….taking age into account.
Copyright American Society of Nuclear Cardiology

## Slide 3
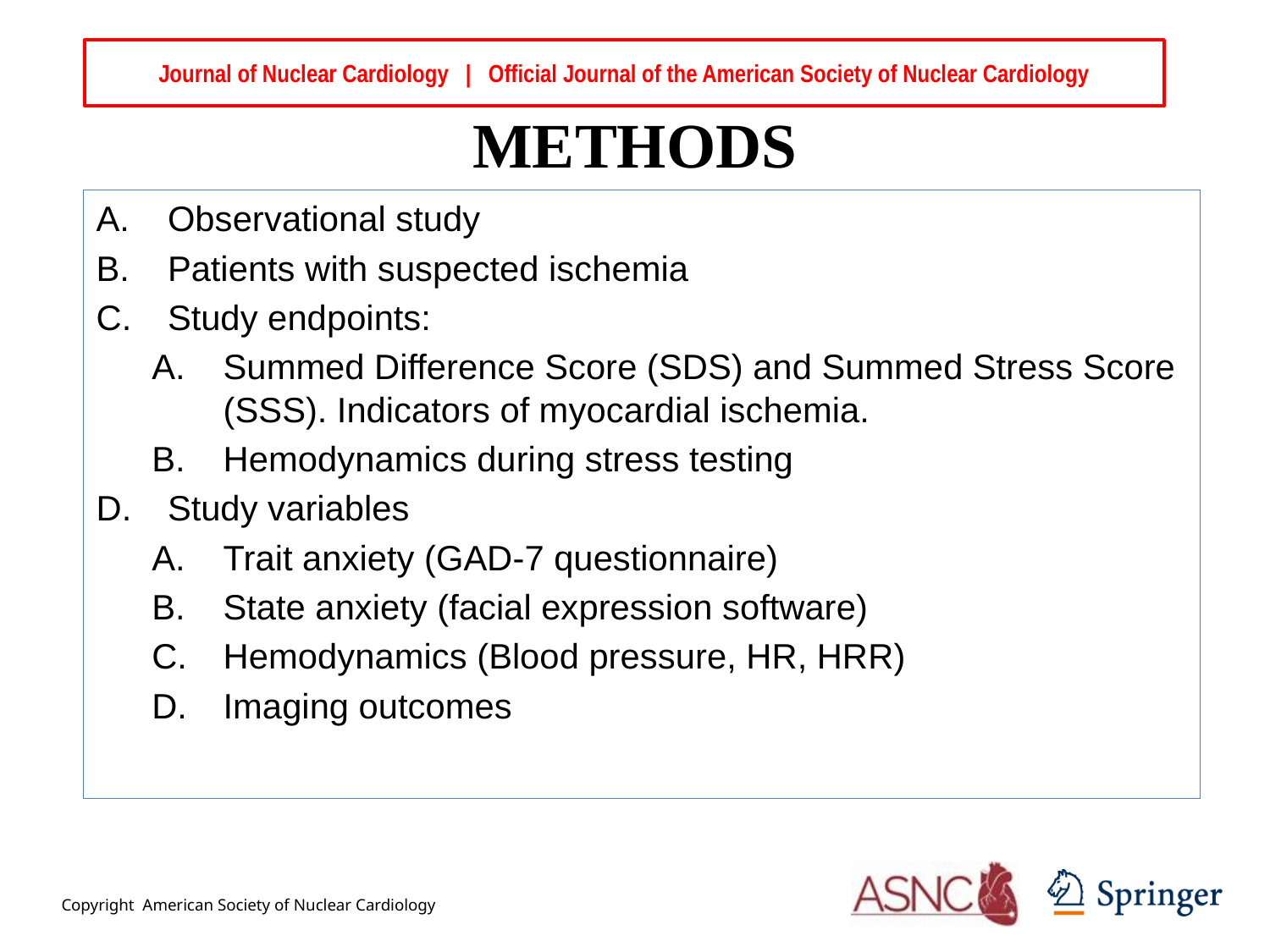

Journal of Nuclear Cardiology | Official Journal of the American Society of Nuclear Cardiology
# METHODS
Observational study
Patients with suspected ischemia
Study endpoints:
Summed Difference Score (SDS) and Summed Stress Score (SSS). Indicators of myocardial ischemia.
Hemodynamics during stress testing
Study variables
Trait anxiety (GAD-7 questionnaire)
State anxiety (facial expression software)
Hemodynamics (Blood pressure, HR, HRR)
Imaging outcomes
Copyright American Society of Nuclear Cardiology

## Slide 4
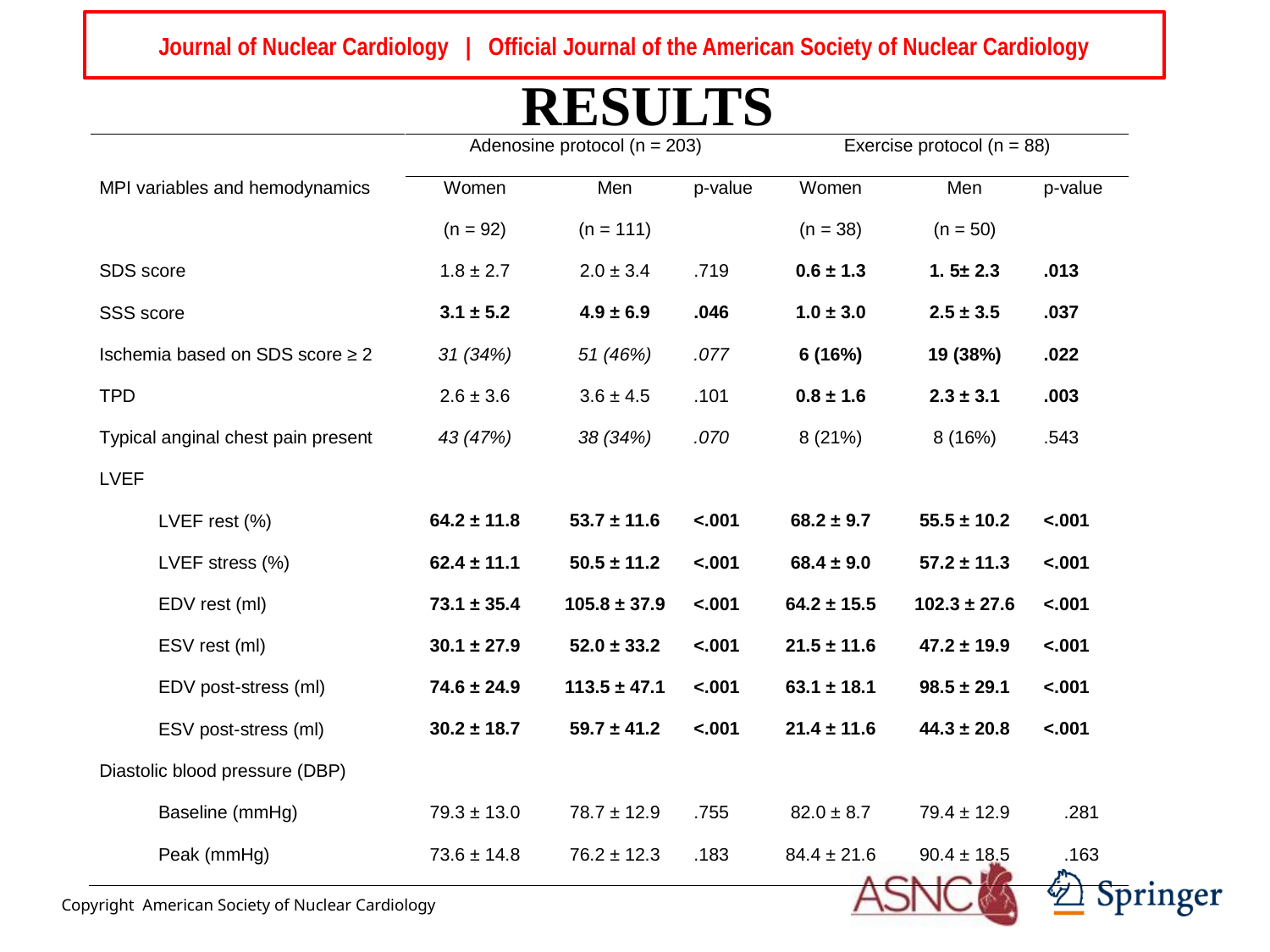

Journal of Nuclear Cardiology | Official Journal of the American Society of Nuclear Cardiology
# RESULTS
Copyright American Society of Nuclear Cardiology

## Slide 5
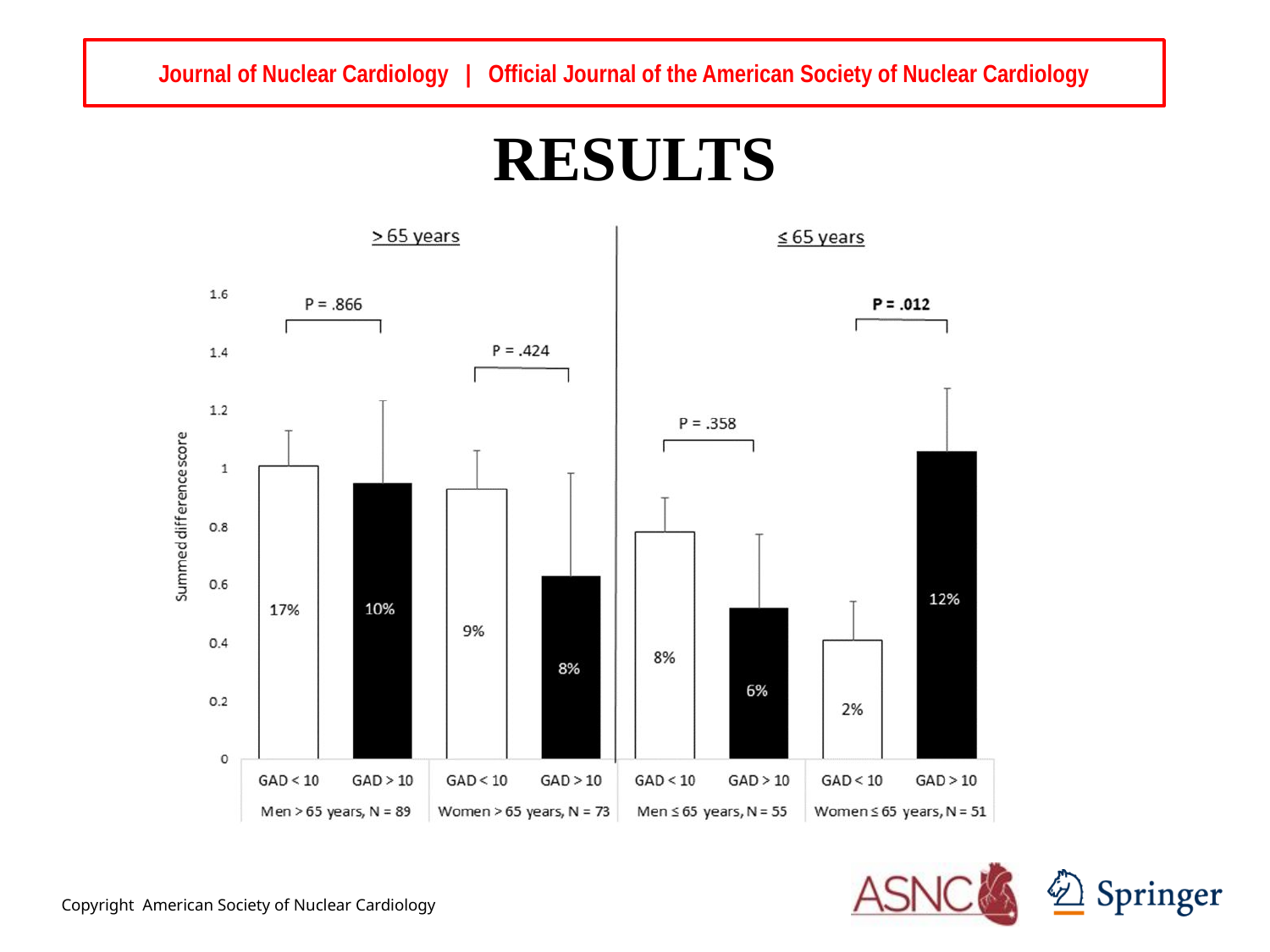

Journal of Nuclear Cardiology | Official Journal of the American Society of Nuclear Cardiology
# RESULTS
Copyright American Society of Nuclear Cardiology

## Slide 6
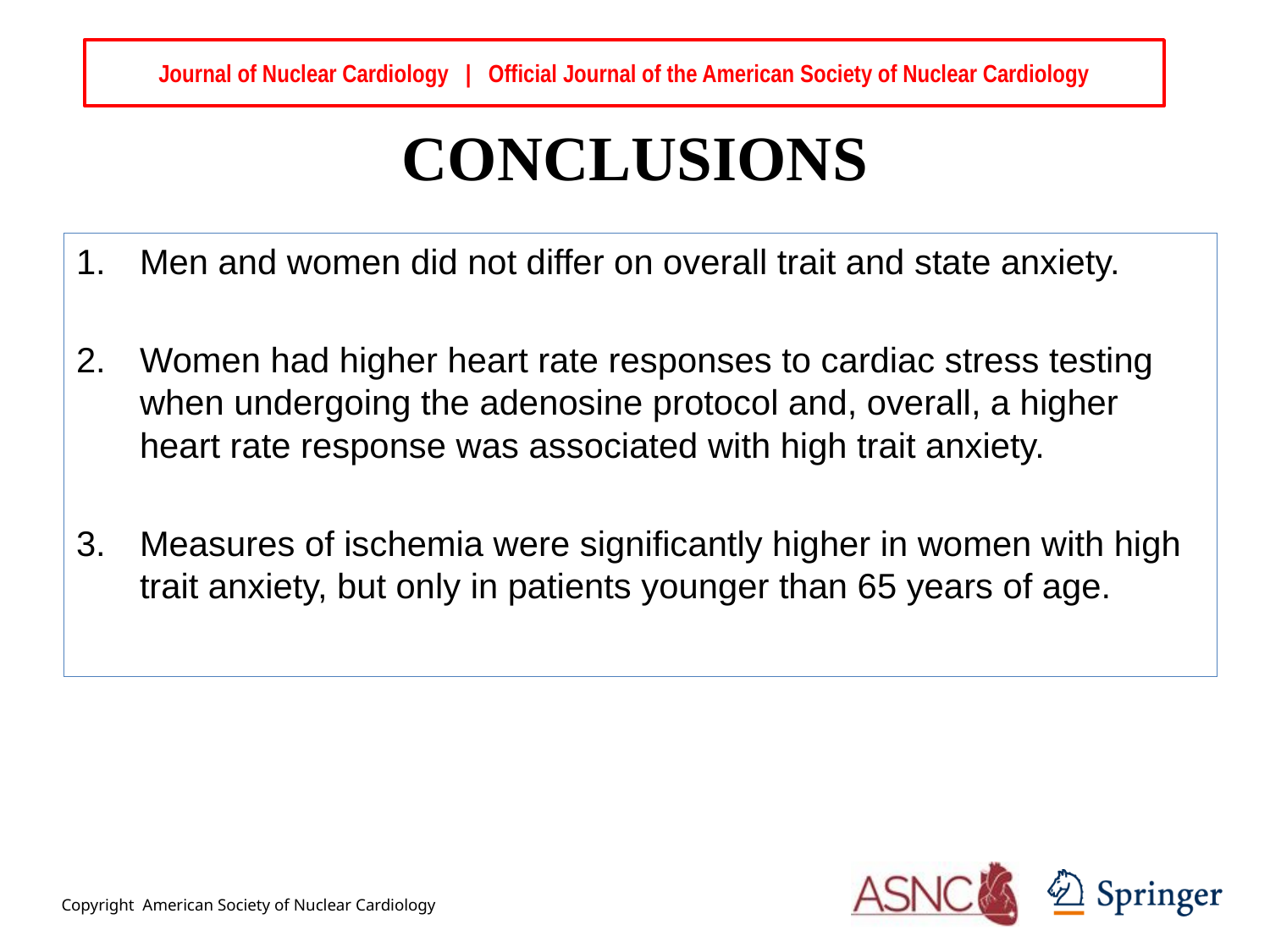

Journal of Nuclear Cardiology | Official Journal of the American Society of Nuclear Cardiology
# CONCLUSIONS
Men and women did not differ on overall trait and state anxiety.
Women had higher heart rate responses to cardiac stress testing when undergoing the adenosine protocol and, overall, a higher heart rate response was associated with high trait anxiety.
Measures of ischemia were significantly higher in women with high trait anxiety, but only in patients younger than 65 years of age.
Copyright American Society of Nuclear Cardiology
